# Alteration of Gut Microbiota in Carbapenem-Resistant Enterobacteriaceae Carriers during Fecal Microbiota Transplantation According to Decolonization Periods

**DOI:** 10.3390/microorganisms9020352

**Published:** 2021-02-10

**Authors:** Jin-Jae Lee, Dongeun Yong, Ki Tae Suk, Dong Joon Kim, Heung-Jeong Woo, Seung Soon Lee, Bong-Soo Kim

**Affiliations:** 1 Department of Life Science and Multidisciplinary Genome Institute, Hallym University, Chuncheon 24252, Korea; ljj807@hallym.ac.kr; 2 Institute for Liver and Digestive Diseases, Hallym University, Chuncheon 24252, Korea; ktsuk@hallym.ac.kr (K.T.S.); djkim@hallym.ac.kr (D.J.K.); 3 Department of Laboratory Medicine and Research Institute of Bacterial Resistance, Yonsei University College of Medicine, Seoul 03722, Korea; DEYONG@yuhs.ac; 4 Department of Internal Medicine, Division of Gastroenterology and Hepatology, Hallym University, Chuncheon Sacred Heart Hospital, Chuncheon 24253, Korea; 5 Department of Internal Medicine, Division of Infectious Diseases, Hallym University Dongtan Sacred Heart Hospital, Hallym University College of Medicine, Hwaseong 18450, Korea; infwoo@hallym.or.kr; 6 Department of Internal Medicine, Division of Infectious Diseases, Hallym University Chuncheon Sacred Heart Hospital, Hallym University College of Medicine, Chuncheon 24252, Korea

**Keywords:** carbapenem-resistant *Enterobacteriaceae* (CRE), fecal microbiota transplantation (FMT), gut microbiota, decolonization

## Abstract

Fecal microbiota transplantation (FMT) has been suggested as an alternative therapeutic option to decolonize carbapenem-resistant *Enterobacteriaceae* (CRE). However, the analysis of gut microbiota alteration in CRE carriers during FMT is still limited. Here, gut microbiota changes in CRE carriers were evaluated during FMT according to decolonization periods. The decolonization of 10 CRE carriers was evaluated after FMT, using serial consecutive rectal swab cultures. Alterations of gut microbiota before and after FMT (56 serial samples) were analyzed using high-throughput sequencing. The decolonization rates of CRE carriers were 40%, 50%, and 90% within 1, 3 and 5 months after initial FMT, respectively. Gut microbiota significantly changed after FMT (*p* = 0.003). Microbiota alteration was different between the early decolonization carriers (EDC) and late decolonization carriers (LDC). Microbiota convergence in carriers to donors was detected in EDC within 4 weeks, and keystone genera within the Bacteroidetes were found in the gut microbiota of EDC before FMT. The relative abundance of *Klebsiella* was lower in EDC than in LDC, before and after FMT. Our results indicate that FMT is a potential option for CRE decolonization. The gut microbiota of CRE carriers could be used to predict decolonization timing after FMT, and determine repeated FMT necessity.

## 1. Introduction

Carbapenem-resistant *Enterobacteriaceae* (CRE) is an urgent antibiotic threat due to high infection-associated mortality, limited therapeutic options, and potential to rapidly spread between bacterial species [1,2]. In the healthcare setting, the carriage rates of extended spectrum beta-lactamase (ESBL) or CRE were 75.2% at 3 months, 55.3% at 6 months, and 35.2% at 12 months, after initial identification [3]. Although short-term antibiotic therapy for the decolonization of antibiotic-resistant bacteria (ARB) could reduce carriage during therapy, its long-term effects are unclear [3]. Pathogens can acquire resistance to antibiotics, and side effects of the antibiotics on gut microbiota, including increased susceptibility to a range of infections through inevitable microbiota dysbiosis, have been reported [4,5]. Therefore, alternative solutions to reduce or eradicate ARB in patients are necessary.

Gut microbiota plays important roles in metabolic processes, immune modulation, and protection against pathogen colonization. As microbiota dysbiosis can be linked to function loss, microbiota engraftment from healthy donors can recover these functions. Fecal microbiota transplantation (FMT) has become a popular approach to microbiota modulation and has been widely used as a treatment option for multiple recurrences of *Clostridioides difficile* infection (CDI), with a substantial reduction in antibiotic resistance genes as well as gut microbiota changes [6,7]. A high response rate to FMT in CDI treatment has led to applications in various diseases, including ulcerative colitis, Crohn’s disease, and multiple antibiotic resistance [8,9,10]. The prevalence of *Enterobacteriaceae* with multiple resistance to beta-lactam antibiotics, including carbapenems, vancomycin-resistant *Enterococcus* (VRE) and methicillin-resistant *Staphylococcus aureus* (MRSA), could be reduced by FMT [11]. FMT’s efficacy in decolonizing multidrug-resistant (MDR) organisms and preventing recurrent MDR infections was 37.5 to 87.5% in a previous study [10]. CRE carriers showed decreased diversity in gut microbiota and increased relative abundance of *Enterobacteriaceae* [12]. Therefore, FMT could be an attractive option to reduce CRE by gut microbiota modulation. However, the analysis of gut microbiota alteration in CRE carriers during FMT is still limited.

To understand the influence of indigenous gut microbiota on the decolonization of CRE and the alteration of microbiota during FMT, we analyzed the longitudinal changes in gut microbiota from 10 carbapenemase-producing (CP-) CRE carriers during FMT treatment over the stipulated decolonization periods.

## 2. Materials and Methods

### 2.1. Subject Selection and FMT Procedures

CP-CRE carriers (≥18 years old) with one or more of the following risk factors for prolonged carriage of CP-CRE were selected for FMT treatment at Hallym University Chuncheon Sacred Heart Hospital: (1) Carbapenem use for >3 d after CP-CRE identification; (2) Positive CP-CRE in clinical specimens, defined as every clinical specimen positive for CP-CRE other than rectal swab, regardless of clinical signs of infection; (3) The presence of concurrent CDI after CP-CRE identification; (4) Long duration of hospitalization (>2 months) after CP-CRE acquisition [13]. Prolonged carriage of CP-CRE was defined as the identification of CRE in either a rectal swab or clinical culture for >3 months after initial detection.

Frozen or capsulized gastrointestinal microbiota (MicroBiotix, Inc., Seoul, Korea) from unrelated pre-screened healthy donors were supplied on the day of each FMT after informed consent was provided. The human microbiota banking project of the Microbiotix Corporation, a non-profit human microbiota bank for FMT, was approved by the Severance Hospital Institutional Review Board, Seoul, Korea (IRB no. 4-2016-0871). Screening tests for donors were performed in a two-stage process based on the Korean Transfusion Guidelines and the European and American human microbiota banks’ donor screening protocols [14,15,16,17]. Firstly, an in-person clinical assessment was performed to evaluate the general health and gastrointestinal conditions of donor candidates, as well as any risk factors for transmissible diseases. Secondly, screening for viral, bacterial, and protozoal pathogens, serological/stool screening, a urea breath test, and a chest posteroanterior radiography were performed to exclude donors with potentially transmissible pathogens.

Antibiotic administration was stopped at least 48 h before performing FMT. A bowel lavage was performed a day prior to FMT. A frozen fecal microbiota preparation was thawed in a water bath at 30 °C for 2 h prior to FMT. Thawed FMT preparations were divided into five 50 mL syringes for administration via a colonoscopy or esophagogastroduodenoscopy. The FMT was mainly performed at the distal end of the ileum or ascending colon via a colonoscopy. If bowel preparation was not performed correctly or entry of the colonoscope was not smooth, FMT was performed at the duodenum by switching to esophagogastroduodenoscopy. Capsulized FMT was performed on one patient who failed to decolonize CRE, even in the second FMT. Twenty pills of capsulized stool were administered to the patient along with cranberry juice daily, for 2 consecutive days. Rectal swabs were collected before FMT and once a week after FMT treatment and stored at −80 °C until further use.

Decolonization of CP-CRE was defined as three consecutive CRE-negative rectal swab cultures repeated with a 3 d interval after FMT. The decolonization time of CP-CRE after FMT was defined as the time interval from the initial time point of FMT until the time point of three consecutive CRE-negative surveillance rectal swab cultures, as well as clinical cultures. The study protocol was approved by the Institutional Review Board at Hallym University Chuncheon Sacred Heart Hospital (IRB no. 2019-05-012 and 2020-08-006) and was registered on the ClinicalTrials.gov public website (no. NCT04583098). Written informed consent for participation in this study was obtained from all patients. The study complies with the Declaration of Helsinki.

### 2.2. Culture Assay and Polymerase Chain Reaction (PCR) for CRE Detection in Carriers

Specimens including rectal swabs were collected from patients during the study period. Rectal swabs were plated on primary CHROMagar KPC medium (CHROMagar, Paris, France) for CRE screening. Other clinical specimens such as sputum, urine, and blood were plated on blood agar (Becton-Dickinson, Sparks, MD, USA) and MacConkey agar (Becton-Dickinson). Agar plates were incubated at 36.5 °C for 16–24 h. Taxonomic identification and antibiotic susceptibility tests, including carbapenem for isolates, were performed using the VITEK^®^ 2 system (bioMerieux, Marcy I’Etoile, France) according to the Clinical and Laboratory Standards Institute M100S guidelines [18]. Carbapenemase production in each isolate was analyzed using the CarbaNP test [19]. To validate carbapenamase production in isolates, target genes encoding *bla*_KPC_, *bla*_NDM_, *bla*_IMP_, *bla*_VIM_, and *bla*_OXA-48_ were amplified and confirmed by gel electrophoresis [20].

### 2.3. Quantitative Real-Time PCR and MiSeq Sequencing

Metagenomic DNA was extracted from collected rectal swab samples using the RNeasy PowerMicrobiome RNA Isolation Kit (Qiagen, Hilden, Germany) according to the manufacturer’s instructions. Extracted DNA was verified using 1% agarose gel electrophoresis, and quantification was performed using the BioPhotometer D30 and μCuvette G1.0 (Eppendrof, Hamburg, Germany).

The 16S rRNA gene copies of bacteria in each sample were measured and compared by quantitative real-time PCR, based on the 16S rRNA gene using 340F (5′-TCCTACGGGAGGCAGCAG-3′) and 518R (5′-ATTACCGCGGCTGCTGG-3′) primers on a Thermal Cycler Dice Real Time System III (Takara Bio). Amplifications were conducted in a final volume of 25 µL, containing 12.5 µL 2 × SYBR green premix Ex Taq (Takara Bio, Otsu, Japan), 2 µM of each primer, and 1 µL of DNA template (10-fold serial dilution of DNA) or distilled water (negative control). The reaction conditions consisted of an initial denaturation at 95 °C for 5 min, followed by 40 cycles of 95 °C for 5 s and 60 °C for 30 s. Standard curves were generated from parallel reactions with serial log-concentrations of the copy number of the bacterial 16S rRNA gene from *Escherichia coli* K12 W3110, and each reaction was performed in triplicate.

To analyze the microbiota in samples, metagenomic DNA was amplified following the 16S Metagenomic Sequencing library for the MiSeq system manufacturer’s guidelines (Illumina, San Diego, CA, USA), as described previously [21,22]. Briefly, the V1–V3 hypervariable regions of the 16S rRNA gene were amplified using primers with adapters (forward: 5′-adapter [TCGTCGGCAGCGTCAGATGTGTATAAGAGACAG]- GAGTTTGATCMTGGCTCAG-3′; reverse: 5′-adapter [GTCTCGTGGGCTCGGAGATGTGTATAAGAGACAG]-ATTACCGCGGCTGCTGG-3′). The first step of amplification was performed in a final volume of 25 uL containing 1µM of each primer, 1.25 U *Ex Taq* polymerase (Takara Bio), 2.5 μL *Ex Taq* Buffer (10×), 4 μL dNTP Mix, and 2.5 μL DNA template. Initial denaturation was conducted at 95 °C for 3 min, followed by 25 cycles of denaturation at 95 °C for 30 s, annealing at 55 °C for 30 s, extension at 72 °C for 30 s, and final extension at 72 °C for 5 min. The amplicons were verified with 2% agarose gel electrophoresis, and purification and size selection were performed using the Agencourt AMPure XP beads (Beckman Coulter, Indianapolis, IN, USA). Index PCR reactions were performed using 5 μL purified PCR product in a final volume of 50 μL using the Nextera XT Index Kit (Illumina). The index PCR reaction conditions consisted of initial denaturation at 95 °C for 3 min, followed by 8 cycles of denaturation at 95 °C for 30 s, annealing at 55 °C for 30 s, extension at 72 °C for 30 s, and final extension at 72 °C for 5 min. The PCR products of each sample were purified again using Agencourt AMPure XP beads (Beckman Coulter). The library was quantified using the Takara PCR Thermal Cycler Dice Real Time System III with the GenNext NGS Library Quantification Kit (Toyobo, Osaka, Japan). To detect contamination in each experimental step, negative controls (a blank swab and distilled water) were performed with the same procedures, along with fecal samples. Equimolar concentrations of each library from different samples were pooled and sequenced on the Illumina MiSeq system (300 bp paired ends) according to the manufacturer’s instructions.

### 2.4. Sequencing Data Analysis

The obtained sequence reads were analyzed using the DADA2 R package (ver 1.14.0) [23] and operational taxonomic units (OTUs)-based analyses. The DADA2 microbiome pipeline was used for pre-processing, including quality check, de-noising, merging, and chimera removal. OTUs-based analyses were further analyzed using the amplicon sequence variants (ASVs) table obtained after pre-processing. Briefly, sequence reads were quality-filtered and trimmed using the *filterAndTrim*() function; an expected error threshold of 2, combined with trimming 10 bp from the ends of forward reads and 40 bp from the ends of reverse reads, and short length sequences and ambiguous sequence bases were removed by default parameters of DADA2. Filtered reads were subsequently de-noised by sequencing error rates. A model for sequencing error rates was constructed using the *learnErrors*() function. ASVs were inferred from filtered sequences using the *dada*() function. ASVs from forward and reverse sequences were merged using the *mergePairs*() function, and chimeric ASVs were detected and removed using the *removeBimeraDenove*() function. The fasta file of each sample was extracted from non-chimeric ASVs and the resulting sequences were subsequently clustered into OTUs using a 97% sequence similarity from the CLC Genome Workbench OTU clustering pipeline (ver. 8.5.1, Qiagen). Taxonomic classification of the OTUs was performed using the EzTaxon database [24]. To compare the diversity indices between samples, read numbers were normalized by random subsampling, and diversity indices were calculated using MOTHUR (ver. 1.35.1) [25]. Linear discriminant analysis effect size (LEfSe) was used to identify different genera between groups [26]. Genera with a logarithmic LDA score >2.0 were considered significantly different.

### 2.5. Co-Occurrence Network Analysis

Co-occurrence networks were constructed to explore the interaction patterns among different bacterial taxa, based on the CoNet module in Cytoscape (ver. 3.7.2) [27]. The 30 most abundant genera in each group were selected for the co-occurrence network. Four similarity matrices were calculated using the Pearson and Spearman correlation methods, mutual information similarity method, Bray–Curtis dissimilarity, and Kullback–Leibler dissimilarity distance methods to construct networks. The threshold edge number was set to 100. A bootstrap resampling method with 1000 iterations in an edge score routine was employed to achieve randomization, and a Benjamini–Hochberg multiple test correction (corrected *p* < 0.01) was performed to remove false positives. *p* value merging was achieved using the Brown method to obtain the final co-occurrence networks. Topological parameters were determined by NetworkAnalyzer (ver. 2.7), and keystone genera were selected based on closeness centrality and betweenness centrality values.

### 2.6. Statistical Analysis

Significant differences between groups were calculated using the Mann–Whitney U test in R software (ver. 3.6.1). Non-metric multidimensional scaling (NMDS) was tested using analysis of similarities (ANOSIM) as implemented in the *vegan* R package. Results with *p* < 0.05 or corrected *p* < 0.05 were considered significant. Correlations between bacterial members were calculated by Spearman’s rank correlation coefficient in R software. Indicator genera (*p* < 0.05) were determined using the *labdsv* R package.

## 3. Results

### 3.1. Clinical Features of CP-CRE Carriers

FMT was performed to decolonize the CRE in 10 carriers with the risk of prolonged CP-CRE carriage, and their baseline characteristics are shown in Table 1. The mean age of carriers was 72.7 ± 7.8 years, and seven carriers were female. The average duration of CP-CRE carriage prior to FMT was 5.7 ± 2.1 months. The decolonization rates were 40.0% (4/10), 50.0% (5/10), and 90.0% (9/10) within 1, 3, and 5 months after initial FMT treatment, respectively. The average and median time to successful decolonization after initial FMT were 65.2 ± 4.9 d and 51.0 d, respectively. Despite having risk factors for prolonged CP-CRE carriage, four carriers (40%) showed faster successful decolonization within 1 month after initial FMT than the other carriers. Three carriers showed decolonization after the second FMT, and one carrier showed decolonization after the third FMT treatment. One carrier (no. 7) was not decolonized for 138 d despite the second FMT. The overall decolonization rate was 90.0% (9/10), irrespective of the time point after FMT treatment.

### 3.2. Changes in Gut Microbiota of CP-CRE Carriers after FMT Treatment

CRE decolonization and changes in gut microbiota after FMT were analyzed by both culture assays and 16S rRNA-based sequencing. The sampling times and decolonization day in each carrier are indicated in Figure S1. The detection of carbapenem-resistant *Enterobacteriaceae* by culture during FMT is summarized in Supplementary Table S1. Nine carriers fulfilled the CRE decolonization criteria, and each patient had a different decolonization period.

**Figure 1:**
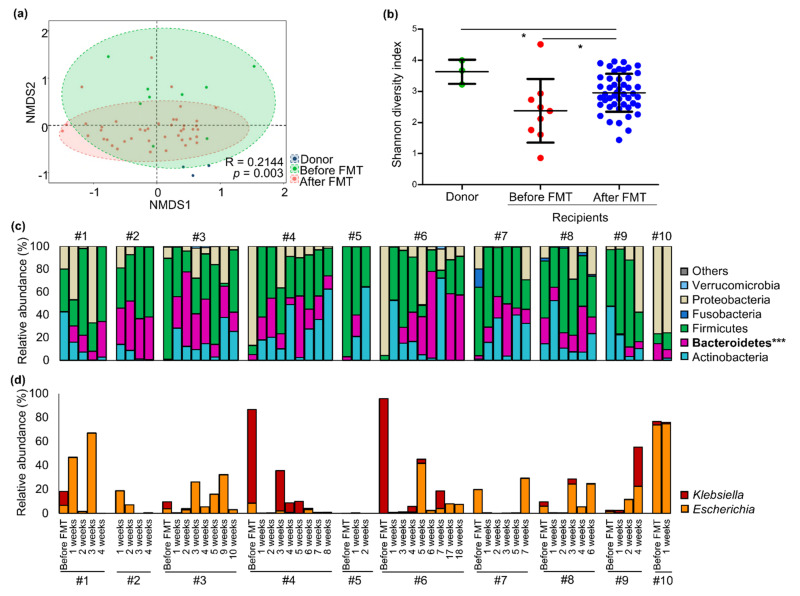
Gut microbiota changes in CRE carriers during FMT treatment. (**a**) The difference in microbiota between carriers and donors were compared in NMDS plots. (**b**) Shannon diversity indices of gut microbiota were compared among donor, carriers before FMT, and carriers after FMT. (**c**) Changes in phylum composition were compared in each carrier according to sampling times. (**d**) Changes in detected CRE genera, *Klebsiella* and *Escherichia*, were compared in each carrier according to sampling times. *** *p* < 0.001, * *p* < 0.05. CRE, carbapenem-resistant Enterobacteriaceae; FMT, fecal microbiota transplantation; NMDS, non-metric multidimensional scaling.

Gut microbiota changes were analyzed in non-metric multidimensional scaling (NMDS) plots based on the Bray–Curtis dissimilarity, and gut microbiota significantly changed after FMT treatment (ANOSIM, *p* = 0.003; Figure 1a). The diversity of gut microbiota in recipients increased to a greater extent after FMT than before FMT (*p* < 0.05; Figure 1b). The differences of gut microbiota in carriers after FMT were unaffected by age and sex (ANOSIM, Age: *p* = 0.308; Sex: *p* = 0.366; Figure S2A), whereas they were significantly related to the follow-up sampling times after FMT treatment (*p* = 0.041; Figure S2B).

The relative abundance of Bacteroidetes significantly increased in the gut microbiota of all carriers after FMT treatment (*p* < 0.001; Figure 1c). Other phyla were otherwise changed among carriers. Genera changes were compared via times after FMT (Figure S3). Although the changed genera were different in terms of times after FMT in each carrier, more diverse genera were detected after FMT than before FMT. The relative abundances of the CRE genera, *Klebsiella* and *Escherichia*, were compared along times in each carrier (Figure 1d). Although their changes fluctuated in terms of follow-up times, the relative abundances of *Klebsiella* were reduced after FMT. However, the proportions of *Escherichia* were maintained in some carriers. These results were consistent with those of culture assays (Table S1).

### 3.3. Comparison of Gut Microbiota between Early Decolonization Carriers and Late Decolonization Carriers

**Figure 2:**
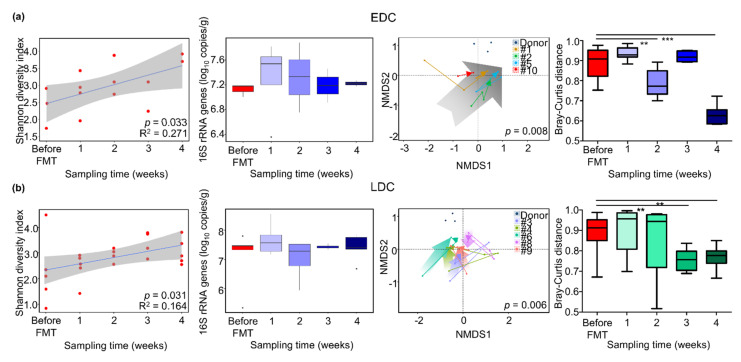
The alterations of gut microbiota were compared between (**a**) the early decolonization carriers (EDC) and (**b**) late decolonization carriers (LDC) in terms of sampling times. The changes in diversity were compared using the Shannon diversity index in terms of times. Bacterial 16S rRNA gene copies were estimated by quantitative real-time PCR. The convergence of gut microbiota in carriers to donor microbiota was analyzed using NMDS plots. The differences in gut microbiota via times were determined by the Bray–Curtis dissimilarity. *** *p* < 0.001, ** *p* < 0.01. NMDS, non-metric multidimensional scaling.

As four carriers (no. 1, 2, 5, and 10) showed decolonization of CP-CRE within 4 weeks, we compared the gut microbiota between these early decolonization carriers (EDC) and late decolonization carriers (LDC; no. 3, 4, 6, 8, and 9) in terms of times (Figure 2). Although the diversity of gut microbiota was significantly increased in both groups (*p* < 0.05), the increased diversity was higher in the EDC group in terms of time after FMT (R^2^ = 0.271) than in the LDC group (R^2^ = 0.164). Bacterial 16S rRNA gene copies increased after FMT; however, the changes in both groups were not statistically significant (*p* > 0.05). In the EDC group, the convergence of recipient gut microbiota to donor microbiota was found within 4 weeks. The dissimilarity of gut microbiota between recipients and donors decreased within 4 weeks after FMT (*p* < 0.001 compared to before FMT). However, the shifts of gut microbiota in the LDC group varied, and convergence to donor microbiota was not found within 4 weeks. The dissimilarity between gut microbiota in LDC carriers and donors also decreased (*p* < 0.01); however, the distance was higher than that of the EDC group after 4 weeks. Although the distances of gut microbiota between carriers in the LDC group and the donor were higher than those in the EDC group, the distances also decreased after 9 weeks in the LDC group (*p* < 0.01 after 18 weeks compared to before FMT; Figure S4). Gut microbiota diversity in the LDC group increased after 5 weeks; however, changes in bacterial 16S rRNA gene copies were not statistically significant (*p* > 0.05). The relative abundances of Bacteroidetes in the EDC group were higher than those in the LDC group, and the relative abundances of Firmicutes in the EDC group were higher before FMT and lower after FMT than those in the LDC group (Figure S5). Diversity was higher in the EDC group than in the LDC group, before and after FMT treatments. However, these differences were not statistically significant (*p* > 0.05).

**Figure 3:**
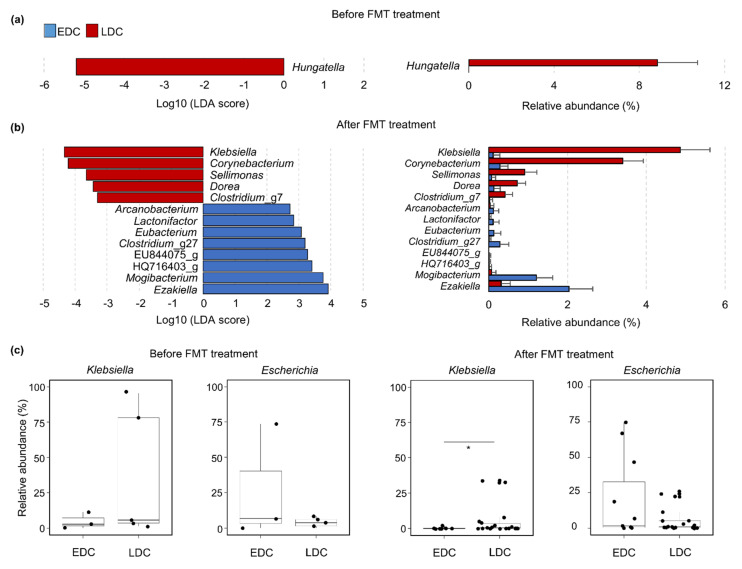
Significantly different genera between the EDC and LDC groups (**a**) before FMT and (**b**) within 4 weeks after FMT were determined by LEfSe analysis. The relative abundances of different genera were compared between the EDC and LDC groups. Genera with a logarithmic LDA score >2.0 were selected as significantly different genera. (**c**) Relative abundances of *Klebsiella* and *Escherichia* were compared between the EDC and LDC groups before and after FMT treatments. * *p* < 0.05. EDC, early decolonization; LDC, late decolonization; FMT, fecal microbiota transplantation; LEfSe, linear discriminant analysis effect size; LDA, linear discriminant analysis.

The difference in gut microbiota between the EDC and LDC groups before and within 4 weeks after FMT was analyzed using LEfSe (Figure 3). Frequently detected genera (>1% of microbiota in each group) were compared between groups. *Hungatella* was significantly different between the EDC and LDC groups before FMT, whereas 13 genera were different between the two groups after FMT treatments. *Hungatella* was only detected in the LDC group. The relative abundances of *Klebsiella*, *Corynebacterium*, *Sellimonas*, *Dorea*, and *Clostridium*_g7 were significantly higher in the LDC group after FMT than those in the EDC group, whereas relative abundances of *Ezakiella*, *Mogibacterium*, uncultured *Christensenellaceae* (HQ716403_g), uncultured *Oscillospiraceae* (EU844075_g), *Clostridium*_g27, *Eubacterium*, *Lactonifactor*, and *Arcanobacterium* were higher in the EDC group compared to the LDC group. The detected CRE genera, *Klebsiella* and *Escherichia*, were compared between the EDC and LDC groups according to FMT treatments. The relative abundances of these genera decreased in both groups after FMT. However, the relative abundance of *Klebsiella* was higher in the LDC group than in the EDC group (*p* < 0.05 after FMT), and the proportion of *Escherichia* was lower in the LDC group than in the EDC group before and after FMT.

### 3.4. Different Alteration of Gut Microbiota along Follow-Up Times between EDC and LDC within 4 Weeks after FMT

The alteration in microbiota between the EDC and LDC groups was compared by indicator genera via times (selected by *p* < 0.05), within 4 weeks after FMT (Figure S6). Three genera (*Anaerococcus*, *Ezakiella*, and *Peptoniphilus*) were significantly changed along times in the EDC group. The relative abundances of these genera increased within 1 week after FMT. In the LDC group, more diverse genera (16 genera) significantly shifted along time after FMT. *Klebsiella* and *Hungatella* were significantly detected before FMT, and their proportions increased again after 3 weeks of FMT. The other 14 genera were significantly detected after FMT. *Peptoniphilus* was a commonly detected genus in both groups after FMT.

**Figure 4:**
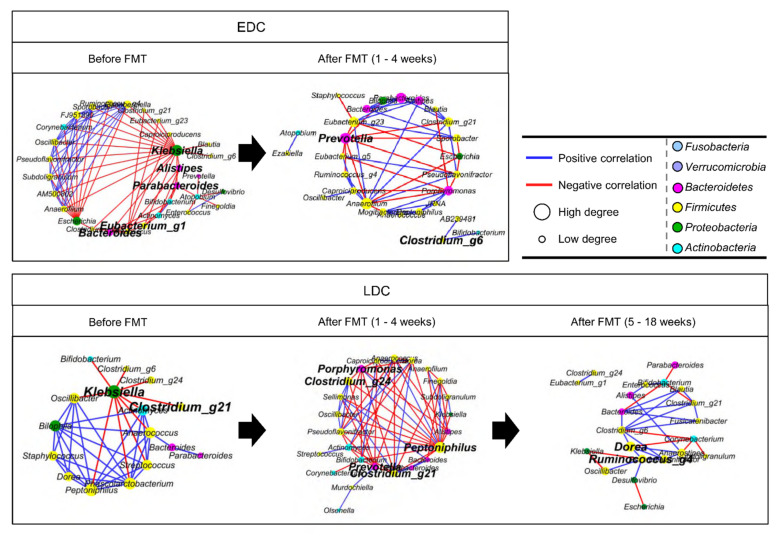
Correlations of genera in gut microbiota were compared between the EDC and LDC groups before and after FMT treatments using co-occurrence network analysis (corrected *p* < 0.01). Bold letters indicate keystone genera. Keystone genera were selected based on closeness centrality and betweenness centrality values. Circle size indicates the degree. Blue line: positive correlation; red line: negative correlation. EDC, early decolonization; LDC, late decolonization; FMT, fecal microbiota transplantation.

Correlations of gut microbiota were compared between the EDC and LDC groups before and after FMT treatments using co-occurrence network analysis (Figure 4). Prior to FMT, the keystone genera were different between EDC and LDC. *Klebsiella* was the common keystone genus in both groups before FMT. However, other keystone genera (*Alistipes*, *Bacteroides*, *Parabacteroides*, *Eubacterium*_g1 in EDC and *Clostridium*_g21 in LDC) were different between the two groups before FMT. Within 4 weeks after FMT, *Prevotella* was a common keystone genus in both groups, whereas *Peptoniphilus* and *Porpyromonas* were unique keystone genera in the LDC group. Interactions between genera in the co-occurrence network were more complex in the LDC group than in the EDC group after FMT.

## 4. Discussion

In this study, 90% (9/10) of CRE carriers showed CRE decolonization within 5 months after FMT treatments, and 40% (4/10) of carriers showed earlier decolonization (within 1 month after FMT) than other carriers regardless of the origin of donor feces (Table S2). The composition and diversity of microbiota significantly changed and the relative abundance of Bacteroidetes significantly increased after FMT, with decreasing proportions of CRE genera. However, alterations in gut microbiota were different between the EDC and LDC groups. The convergence of microbiota in carriers to donor was detected in the EDC group within 4 weeks, and different keystone genera were found before and after FMT between groups. The relative abundance of *Klebsiella* was higher in the LDC group than in the EDC group before and after FMT.

The diversity of gut microbiota significantly increased, and microbiota significantly changed, after FMT. These results are in agreement with previous studies that have shown lower diversity in CRE carriers than in healthy individuals, and increased diversity after FMT treatments [12,28]. Although the relative abundances of *Klebsiella* and *Escherichia* were variable among carriers, their enrichment was found in gut microbiota before FMT. As CRE carriers received antibiotic treatment, this treatment could be a reason for the enrichment of these genera as reported in a previous study, which suggested that antibiotics promote intestinal colonization by *Enterobacteriaceae* [29]. However, their relative abundances decreased, and the Bacteroidetes significantly increased in most carriers, after FMT in the present study. This indicates that FMT can be an alternative option for the decolonization of CRE based on gut microbiota modulation.

Although gut microbiota significantly changed in all carriers after FMT, their alterations were different between the EDC and LDC groups. Previous studies reported the convergence of recipient microbiota towards donor microbiota following FMT treatment [30,31]. Notably, we observed that the convergence towards donor microbiota was found in EDC within 4 weeks. This could be due to the gut microbiota of the donor or the difference in indigenous microbiota between the EDC and LDC groups. Although the EDC and LDC were not categorized by the origin of the donor feces, the efficiency of decolonization was different according to donor (Table S2). This suggest that the donor microbiota could partially influence the decolonization of CRE [32]. *Hungatella* was significantly higher in LDC than in EDC prior to FMT, and five genera were higher in LDC after FMT. Eight genera, including *Ezakiella* and *Mogibacterium*, were higher in the EDC group than in the LDC group after FMT. Although these genera were reported as ARB [33,34,35], significantly abundant genera in the EDC group could have positive effects on gut environment. The loss of *Ezakiella* was detected in sepsis patients [36]; *Mogibacterium* was positively correlated with the short-chain fatty acids concentrations [37]; *Eubacterium* can utilize acetate and lactate to produce butyrate and propionate in the intestine [38]. In particular, the relative abundance of *Klebsiella* was higher in the LDC group before and after FMT, whereas *Escherichia* was higher in the EDC group. Although the relative abundances of *Klebsiella* decreased 1–2 weeks after FMT in the LDC group, the proportion increased again after 3–4 weeks. Therefore, CRE carriers in the LDC group did not decolonize within 4 weeks. This indicated that the high abundance of *Klebsiella* could be related to the late decolonization or the necessity of repeated FMT for CRE decolonization.

Co-occurrence network analysis showed that different gut microbiota between groups before FMT could affect the alteration of microbiota after FMT. *Klebsiella* was a common keystone genus in both groups before FMT; however, more genera negatively correlated with *Klebsiella* in EDC than LDC. In particular, the genera within Bacteroidetes (*Alistipes*, *Bacteroides*, and *Parabacteroides*), which were keystone genera in EDC before FMT, negatively correlated with *Klebsiella*. However, the keystone genus (*Clostridium*) within Firmicutes negatively correlated with *Klebsiella* in the LDC group. Keystone genera are important for maintaining the structure, diversity, and function of the ecosystem through biotic interactions with other members [39]. *Bacteroides* spp. demonstrated that they have the capacity to decompose polysaccharides and produce beneficial metabolites [40]. In addition, Bacteroidetes can prevent the colonization of *K. pneumoniae* in the gut by fortifying the gut immune barrier via IL-36 and macrophages [41]. These results indicated that the gut microbiota was different between the EDC and LDC groups prior to FMT, and this difference could affect the timing of decolonization after FMT. Although *Prevotella* was a common keystone genus in both groups within 4 weeks after FMT, more complex interactions and more keystone genera were detected in the LDC group compared to the EDC group. In a previous study, *Prevotellaceae* was reported as a faster colonizer after FMT, and the colonization of *Prevotella* spp. could induce metabolic changes in microbiota, which reduced IL-18 production and consequently exacerbated intestinal inflammation [42,43]. Thus, *Prevotella* was detected as a keystone genus after FMT in both groups, and a significantly shifted genus within 2 weeks after FMT.

FMT treatment has been increasingly used for various disorders, including CDI, ulcerative colitis, and Crohn’s disease. The therapeutic effect of FMT demonstrated the expansion of donor microbiota and defect correction in microbiota composition [44,45]. Various factors, including bacteria, bacteriophages, metabolites, and extracellular vesicles, in donor feces could influence the modulation of the gut microbiota and the decolonization of CRE in recipients [46,47,48,49,50,51]. Previous studies have reported the decolonization of ARB, including CRE, ESBL-E, and VRE, using FMT [7,52,53,54]. However, most reports demonstrated that ARB disappeared after FMT for recurrent CDI patients. A few studies focused on the decolonization of CRE in carriers, regardless of CDI, by FMT. The present study has the potential to extend our understanding of ARB by FMT.

The limitations of this study were the relatively small number of carriers and the need to detail microbiota shifts based on antibiotic resistance genes. However, this study analyzed the longitudinal shifts of microbiota after FMT and used CRE carriers regardless of CDI. Further, studies are necessary to analyze the modulation of the gut microbiome ecosystem using whole-metagenomics, metatranscriptomics and metabolomics.

## 5. Conclusions

FMT treatments for CRE carriers showed 90% decolonization in this study. The decolonization period was different for the gut microbiota of carriers before FMT. Genera within Bacteroidetes were keystones in gut microbiota of the EDC group before FMT. The relative abundance of *Klebsiella* was lower in the EDC group than in the LDC group, before and after FMT. These results could be applied to predict the decolonization period after FMT and determine the necessity of repeated FMTs for the decolonization of ARB.

## Figures and Tables

**Table 1:** Summary of Characteristics of Selected CRE Carriers.

Carrier No.	Age	Sex	CRE Type	Duration of CRE Carriage before FMT	Carbapenem Use ≥ 3 days after CRE Carriage	CRE (+) in Clinical Specimen after CRE Carriage	Concurrent *C. difficile* Infection after CRE Carriage	Prolonged Hospitalization ≥2 Months after CRE Carriage	FMT Material	FMT Procedure	Time to Decolonization of CRE from FMT
1	78	Female	KPC-CRE	7 months	+	+	-	+	Unrelated donor, frozen stool	Terminal ileum and ascending colon via colonoscopy	25 days
2	68	Male	KPC-CRE	4 months	+	+	-	+	Unrelated donor, frozen stool	Duodenum via EGD and ascending colon via colonoscopy	26 days
3	80	Female	KPC-CRE	7 months	+	+	-	+	Unrelated donor, frozen stool	Duodenum via EGD (1st and 2nd FMT)	15 days after 2nd FMT (106 days after 1st FMT)
4	79	Male	KPC-CRE	6 months	+	+	-	+	Unrelated donor, frozen stool	Terminal ileum via colonoscopy	51 days
5	75	Female	KPC-CRE (& VRE)	5 months	+	+	+	+	Unrelated donor, frozen stool	Ascending colon via colonoscopy	15 days
6	75	Female	KPC-CRE (& VRE)	4 months	+	+	-	+	Unrelated donor, frozen stool	Terminal ileum via colonoscopy (1st FMT), duodenum via EGD (2nd FMT)	34 days after 2nd FMT (117 days after 1st FMT)
7	57	Male	KPC-CRE	7 months	+	-	-	+	Unrelated donor, frozen stool	Terminal ileum and ascending colon via colonoscopy (1st and 2nd FMT)	not decolonized (followed until 138 days after 1st FMT)
8	81	Female	KPC-CRE	10 months	+	+	+	+	Unrelated donor, frozen and capsulized stool	Duodenum via EGD (1st and 2nd FMT), 20 capsules daily for 2 days (3rd FMT)	16 days after 3rd FMT (137 days after 1st FMT)
9	65	Female	KPC-CRE	3 months	+	-	-	+	Unrelated donor, frozen stool	Terminal ileum via colonoscopy (1st FMT) duodenum via EGD (2nd FMT)	16 days after 2nd FMT (92 days after 1st FMT)
10	69	Female	KPC-CRE	4 months	+	+	-	+	Unrelated donor, frozen stool	Terminal ileum and ascending colon via colonoscopy	18 days

## Data Availability

The sequence reads obtained from this study are available in the EMBL SRA database under the study number PRJEB41057 (http://ebi.ac.uk/ena/data/view/PRJEB41057).

## Supplementary Materials

The following are available online at https://www.mdpi.com/2076-2607/9/2/352/s1, Table S1: Detection of carbapenem-resistant *Enterobacteriaceae* (CRE) in carriers by culture assays during fecal microbiota transplantation (FMT) treatment, Table S2: The origin of donor feces used for fecal microbiota transplantation (FMT) in each carrier, Figure S1: Sampling times and decolonization day for each carrier, Figure S2: The effects of influencing factors on gut microbiota difference, Figure S3: Changes in genus composition during fecal microbiota transplantation (FMT) treatment in each carrier, Figure S4: Alterations of gut microbiota in the late decolonization carriers (LDC) group after 5 weeks, Figure S5: Relative abundances of Bacteroidetes and Firmicutes and microbiota diversity were compared between the early decolonization carriers (EDC) and late decolonization carriers (LDC) groups before and after fecal microbiota transplantation (FMT) treatment, Figure S6: Indicator genera in terms of times were compared between the early decolonization carriers (EDC) and late decolonization carriers (LDC) groups.
