# Supplementary material for: Alteration of Gut Microbiota in Carbapenem-Resistant Enterobacteriaceae Carriers during Fecal Microbiota Transplantation According to Decolonization Periods
Source: Microorganisms. 2021 Feb 10;9(2):352. doi: 10.3390/microorganisms9020352 (PMC7916679; doi:10.3390/microorganisms9020352)

## Supplementary Materials

**Table S1.** Detection of carbapenem-resistant *Enterobacteriaceae* (CRE) in carriers by culture assays during fecal microbiota transplantation (FMT) treatment

| Carrier No. | Isolation of carbapenem-resistant bacteria | Detection periods of carbapenem-resistant bacteria |
|---|---|---|
| 1 | *Klebsiella pneumoniae* | Before FMT to until 2 weeks after FMT |
| 2 | *Klebsiella pneumoniae* | Before FMT to until 3 weeks after FMT |
| 3 | *Klebsiella pneumoniae & Escherichia coli* | Before FMT to until 3 weeks (*K. pneumoniae*) & until 3 weeks (*E. coli*) to 14 weeks after FMT |
| 4 | *Klebsiella pneumoniae & Escherichia coli* | Before FMT (*E. coli*) & until 6 weeks (*K. pneumoniae*) after FMT |
| 5 | *Klebsiella pneumoniae* | Before FMT to until 1 weeks after FMT |
| 6 | *Klebsiella pneumoniae* | Before FMT to until 15 weeks after FMT |
| 7 | *Klebsiella pneumoniae* | Before FMT to until 20 weeks after FMT |
| 8 | *Klebsiella pneumoniae* | Before FMT to until 6 weeks after FMT |
| 9 | *Klebsiella pneumoniae & Enterobacter cloacae* | Before FMT to until 6 weeks after FMT (*K. pneumoniae*) & until 8 weeks (*E. cloacae*) to 12 weeks after FMT |
| 10 | *Klebsiella pneumoniae* | Before FMT to until 1 weeks after FMT |

**Table S2.** The origin of donor feces used for fecal microbiota transplantation (FMT) in each carrier. EDC: early decolonization carriers; LDC: late decolonization carriers

| Donor No. | Carrier No. | Decolonization group |
|---|---|---|
| D1 | 1 | EDC |
| D1 | 2 | EDC |
| D2 | 3 | LDC |
| D2 | 8 | LDC |
| D2 | 10 | EDC |
| D3 | 4 | LDC |
| D3 | 5 | EDC |
| D3 | 6 | LDC |
| D3 | 9 | LDC |
| D4 | 7 | Not decolonized |

**Figure S1.** Sampling times and decolonization day for each carrier

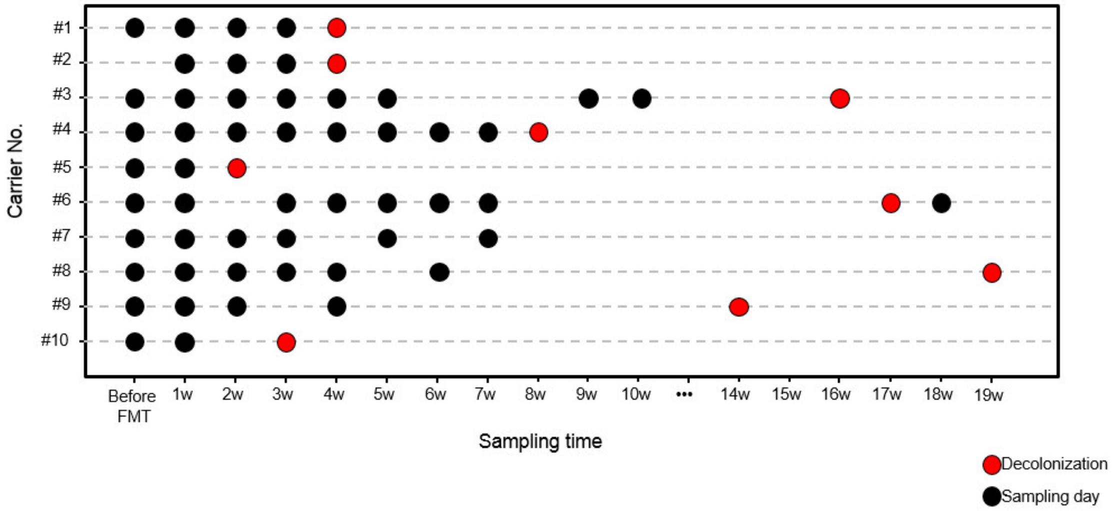

**Figure S2.** The effect of influencing factors on gut microbiota difference. (a) The influence of age and sex on gut microbiota. (b) The effect of sampling times on gut microbiota difference.

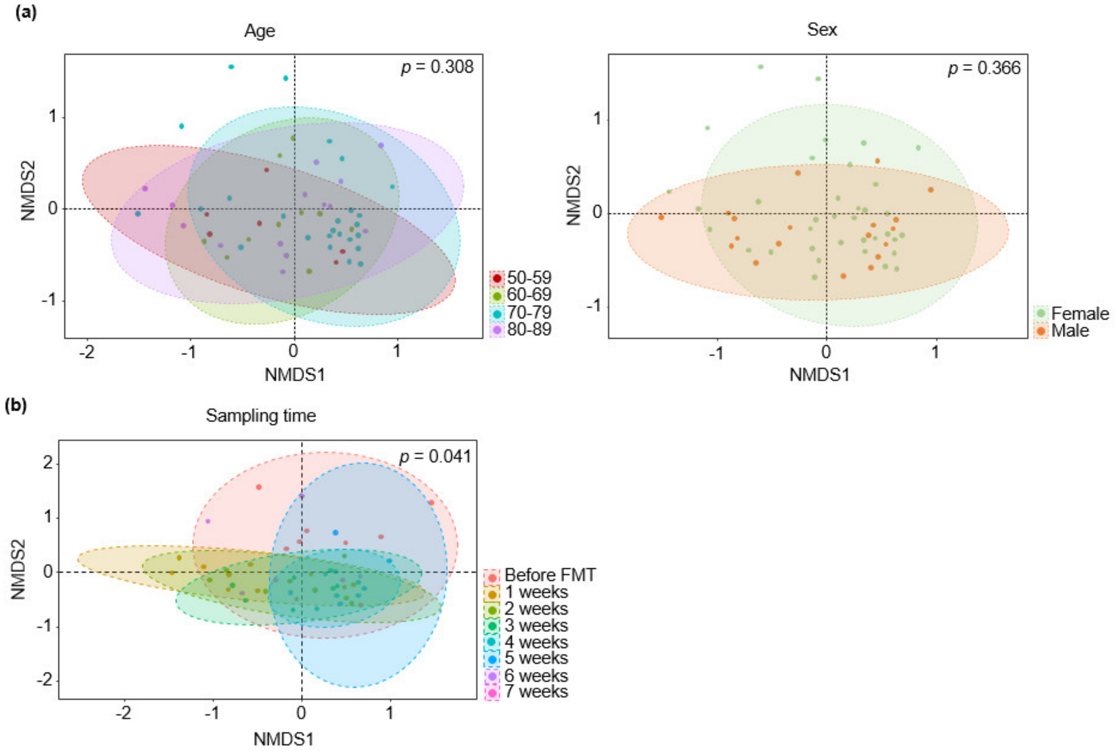

**Figure S3.** Changes in genus composition during fecal microbiota transplantation (FMT) treatment in each carrier.

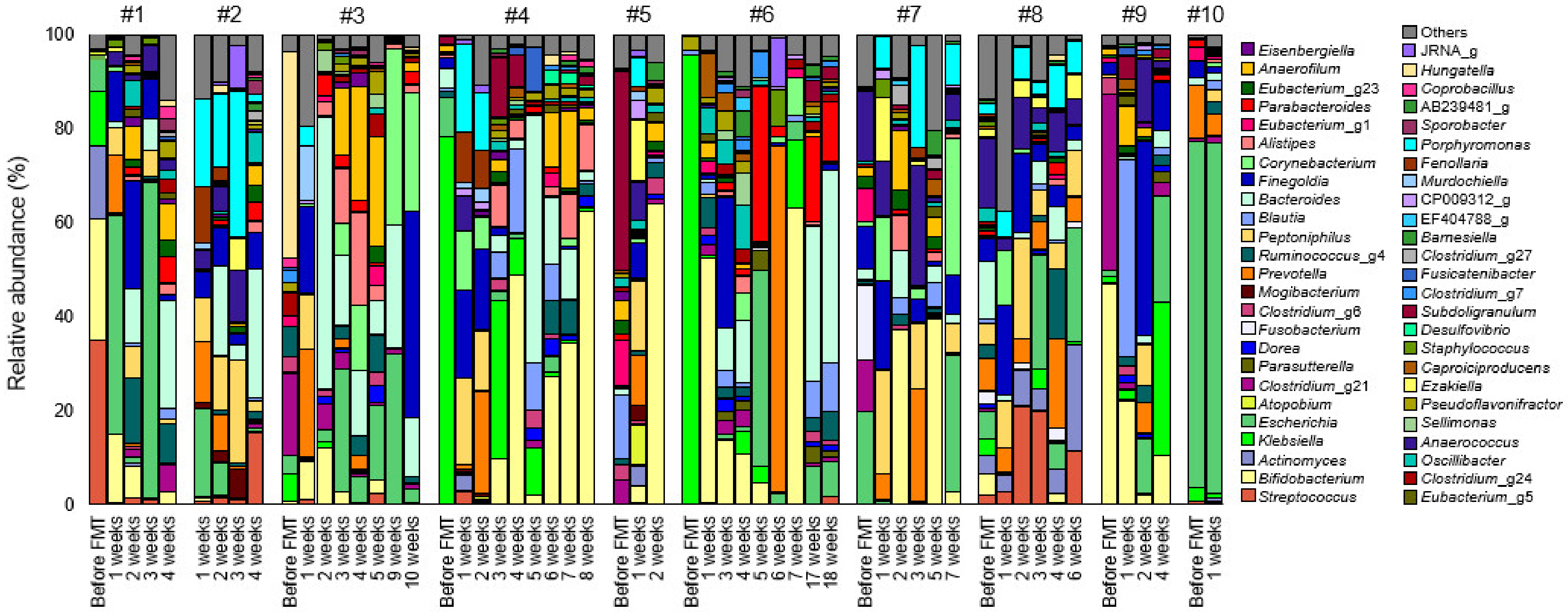

**Figure S4.** Alterations of gut microbiota in the late decolonization carriers (LDC) group after 5 weeks. Diversity was compared using the Shannon diversity index. Bacterial 16S rRNA gene copies were estimated using quantitative real-time PCR. Gut microbiota differences among sampling times were compared using the Bray-Curtis distance. ** $p < 0.01$, * $p < 0.05$.

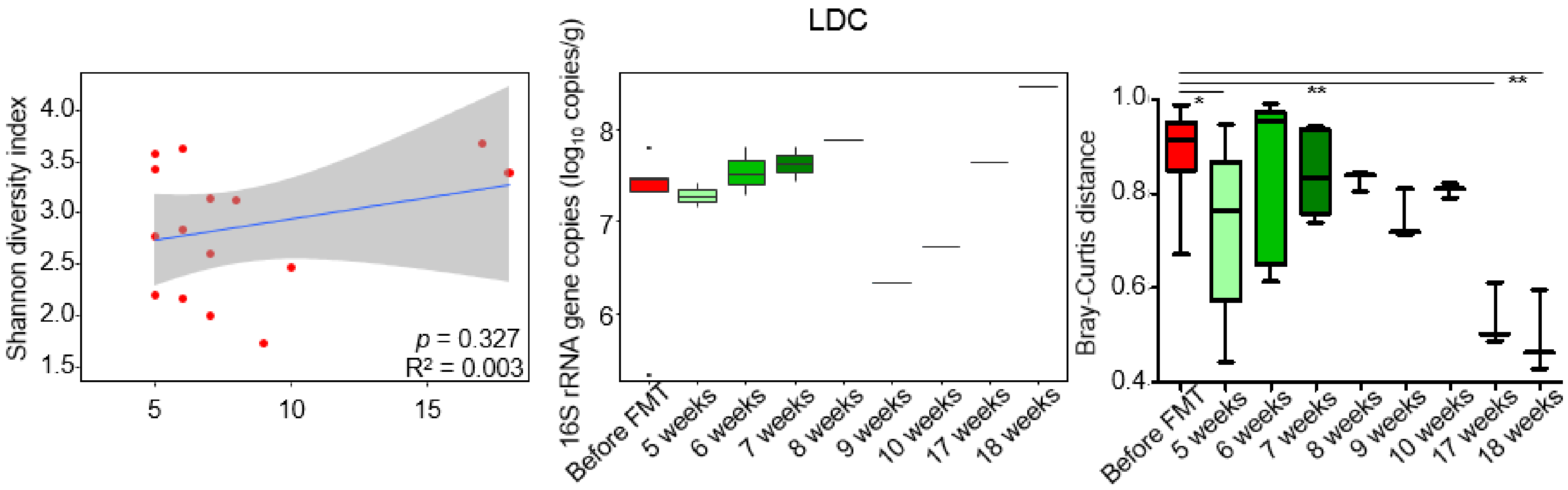

**Figure S5**. Relative abundances of Bacteroidetes and Firmicutes and microbiota diversity were compared between the early decolonization carriers (EDC) and late decolonization carriers (LDC) groups before and after fecal microbiota transplantation (FMT) treatment.

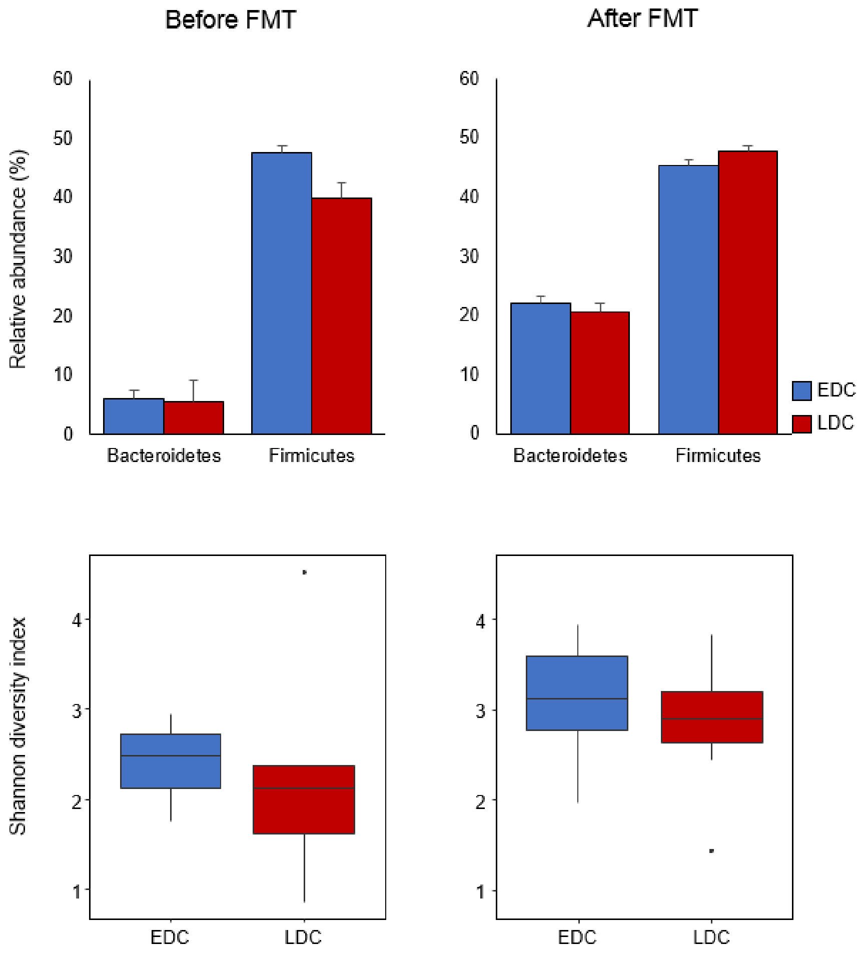

**Figure S6.** Indicator genera along times were compared between the early decolonization carriers (EDC) and late decolonization carriers (LDC) groups. The light blue color represents indicator genera before fecal microbiota transplantation (FMT), and the orange color represents indicator genera after FMT. Circle size represents the relative abundance of the genus. Indicator genera were selected by $p$ value < 0.05.

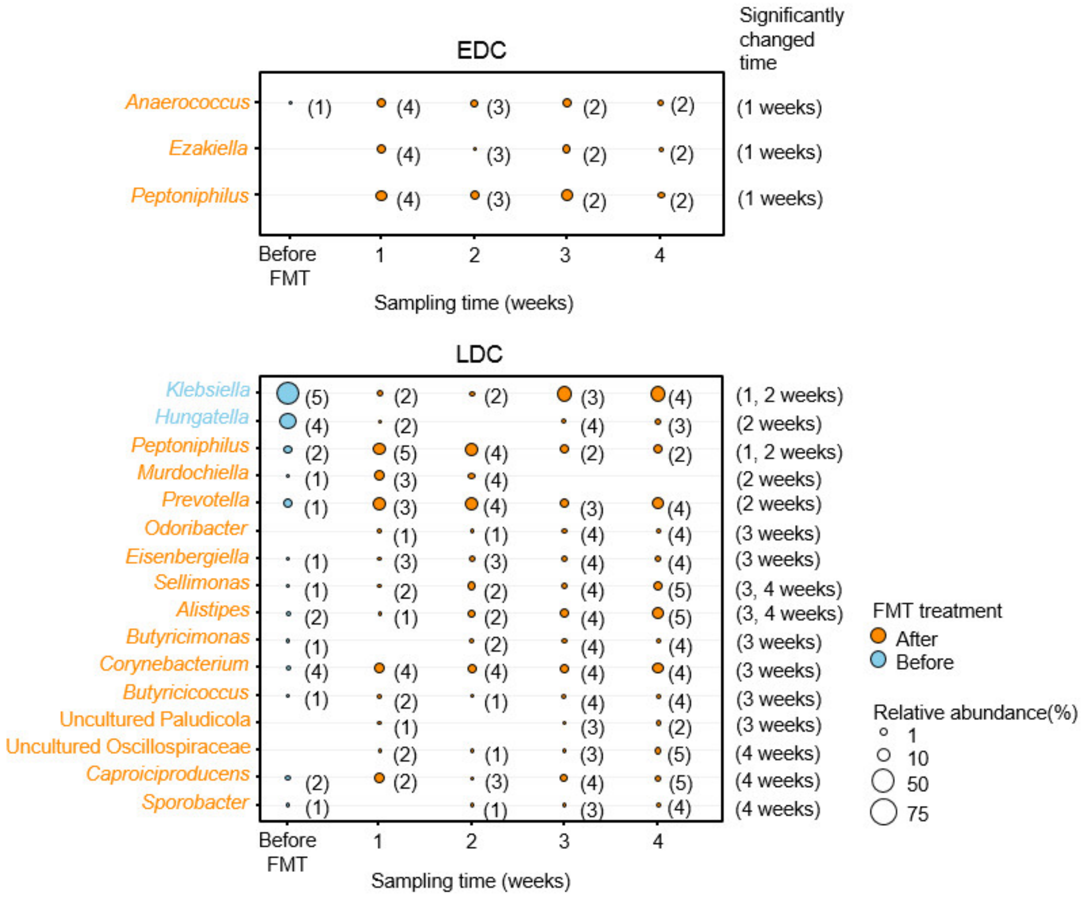

Supplement: Supplementary file 1 [file microorganisms-09-00352-s001.pdf]
